# How much dentists are ethically concerned about overtreatment; a vignette-based survey in Switzerland

**DOI:** 10.1186/s12910-015-0036-6

**Published:** 2015-06-19

**Authors:** Ali Kazemian, Isabelle Berg, Christina Finkel, Shahram Yazdani, Hans-Florian Zeilhofer, Philipp Juergens, Stella Reiter-Theil

**Affiliations:** Department of Community Oral Health, School of Dentistry, Mashhad University of Medical Sciences, Mashhad, Iran; Department of Oral and Cranio-Maxillofacial Surgery, University Hospital Basel, Basel, Switzerland; University Basel, Hightech Research Center, Basel, Switzerland; Educational Development Center (EDC), Shahid Beheshti University of Medical Sciences, Tehran, Iran; Department of Clinical Ethics, Psychiatric Hospital of the University Basel, University Hospital Basel, Basel, Switzerland

**Keywords:** Overtreatment, Undertreatment, Ethical issues, Dental care, Dentists

## Abstract

**Background:**

Overtreatment (or unnecessary treatment) is when medical or dental services are provided with a higher volume or cost than is appropriate. This study aimed to investigate how a group of dentists in Switzerland, a wealthy country known to have high standards of healthcare including dentistry, evaluated the meaning of unnecessary treatments from an ethical perspective and, assessed the expected frequency of different possible behaviors among their peers.

**Methods:**

A vignette describing a situation that is susceptible for overtreatment of a patient was presented to a group of dentists. The vignette was followed by five options. A questionnaire including the vignette was posted to 2482 dentists in the German-speaking areas of Switzerland. The respondents were asked to rate each option according to their estimation about its prevalence and their judgment about the degree to which the behavior is ethically sound.

**Results:**

732 completed questionnaires were returned. According to the responses, the most ethical and the most unethical options are considered to be the most and the least prevalent behaviors among dentists practicing in Switzerland, respectively.

**Conclusions:**

Suggesting unnecessary treatments to patients seems to be an ethically unacceptable conduct in the eyes of a sample of dentists in Switzerland. Although the respondents believed their colleagues were very likely to behave in an ethical way in response to a situation that is susceptible to overtreatment, they still seemed to be concerned about the prevalence of unethical behaviors in this regard.

## Background

For purposes of this study overtreatment is being defined as putting a patient through some medical or surgical procedure when there is little or no evidence that such procedures will improve the patient’s health outcome [1]. It means the use of clinical services having negligible observable benefits, such that demonstrable harm outweighs any minor benefit in virtually all cases [2]. The application of unnecessary, excessive or ineffective medical procedures or drugs is harmful in many patients, especially those who are older and have other illnesses [3, 4] and its cost is enormous [1]. Overtreatment, which is used in this article synonymously for “unnecessary treatment”, has been causatively connected to a variety of health system-oriented or cultural factors such as:

1. fee-for-service systems, which can reward doctors and hospitals for each additional test or procedure performed on patients [5], (overtreatment tends to occur in fee-for-service practices while undertreatment tends to arise in capitation practices) [6];
2. health care marketing by the manufacturers of drugs, diagnostic tests, and medical devices, a foundational responsibility of competitive business ethics and best practices, also contributes to overtreatment [7];
3. the absence, misuse, or misunderstanding of evidence based treatment selection criteria [8];
4. patients or relatives themselves who may assume that more care (more diagnostic tests, more treatments, more procedures) is better care [9]; it is what research suggests as the “conventional wisdom” among most of the public [10, 11];
5. a notion by practitioner’s perspective that it is easier to act than refrain from action and it is difficult to refuse the patients’ wishes [12]; and
6. a lack of specific knowledge/competence among health care professionals [13] including an unawareness or downplay of the ethical literature on diagnostic overload.

Using existing therapies more effectively, the establishment of standards and guidelines and adopting preventive approach to treatment planning seems, currently, to be highly important in preventing overtreatment [4, 14].

Dentistry, as a subcategory of healthcare, is also susceptible to overtreatment or unnecessary treatment. Nevertheless, this issue has not been discussed sufficiently in the literature of dentistry: much of literature on this issue regards the necessity or appropriateness of more emphasis on conservative dental care. Elderton, for example, criticized the fact that many dentists continue to be powered by an aggressive restorative approach which may result in unnecessary treatment and which must now be seen as inappropriate [15]. Without focusing, here, on the ethics of cosmetics as primarily falling under a medical need or a consumer want [16], most experienced dentists may agree that the less that is done to teeth for cosmetic reasons, the lesser are the risks of dissatisfaction, disappointment, or threat of litigation [17]. Ethical aspects of overtreatment in dentistry refer to another important side of the problem. Beside the clinical questions regarding unnecessary treatments, there is additional ethical concern about doing-more-than-what-is-needed. In a study, for example, surgeons reported overtreatment as an ethical dilemma they experienced in deciding the right treatment in different situations [18].

This study aimed to investigate how a selected group of dental professionals in Switzerland evaluated the meaning of unnecessary treatments from an ethical perspective and assessed the frequency of different possible behaviors among their peers. We used a clinical vignette illustrating an ethically meaningful decisional problem to raise practical relevance.

## Methods

In medical and health ethics empirical approaches have gained increasing appreciation: they help to evaluate current practice and enrich the ethical reflection, esp. on matters that display issues of changing or challenging attitudes and actions of agents [19]. This study used a questionnaire-based survey, which contained a series of six static clinical vignettes; each describing a situation on another ethically relevant issue. The following single vignette raises the issue of overtreatment or unnecessary treatment, and was part of the questionnaire used for assessing a broader scope about the ethical judgment of dentists, also regarding the profession. The first draft of the vignette was prepared based on a review of published cases and vignettes on dental ethics, as well as informal discussions with available practicing dentists, and some targeted exploratory interviews [20, 21]. The vignette was located in the private dental office of a hypothetical dentist unfolding a possible problem confronting him/her in the clinical relationship with the patient.

A panel of four oral and maxillofacial surgeons and dental specialists practicing in the University Hospital Basel analyzed the first draft of the vignette and assessed the face validity of the questionnaire. Afterwards, this panel of specialists with their particular interests and experiences generated a list of actually probable behaviors of dentists in accordance to the vignette. They were then asked to state what they expected, as specialists, to be the real responses of typical dentists in Switzerland in this vignette. After merging all the similar proposed behaviors, this generation phase distilled five options to help limit the evaluation of the scenario to manageable and quantifiable categories. Moreover, the specialists including trained ethicists invited to consider these options and make judgments were also asked to judge whether the selected scenario accurately reflects the ethical focus of this study, i.e. overtreatment (unnecessary treatment). The Feasibility of the questionnaire was then tested in a pilot study among a group of 30 dental students in the University Hospital Basel. An interdisciplinary panel of medical and health ethics researchers was invited to analyze the ethical implications of the questions and items.

Respondents were asked to rate each option of the vignette in two ways

1. their estimation about the prevalence of the behavior among dentists in Switzerland (among 100 dentists in Switzerland, how many dentists behave in such a way, on average?);
2. their judgment about the degree to which the behavior is ethically sound (measured on a 7-point Likert scale [22], between dichotomized factors, ‘fully unethical’, and ‘fully ethical’. The neutral position was called ‘ethically neuter’).

Data were actually collected through a mailed self-administered questionnaire. Questionnaires were anonymous, to minimize reporting bias. The envelopes, each containing a questionnaire, a cover letter and a pre-paid return envelope, were posted to 2482 dentists in the German-speaking areas of Switzerland, also members of the Swiss Dental Association. 31 envelopes were rejected due to incorrect or changed addresses. 732 filled questionnaires were returned (29.9 % response rate). No follow-up correspondence was used to increase the response rate or further influence the self-selected responders.

A written statement [22/11/2014] was obtained from Ethical Committee of North-West and Central Switzerland saying that after reviewing the documents, the EKNZ^1^ is able to state that the conduction of this project is not ethically objectionable (cf. Article 51 paragraph 2 federal law of human research) and, thus not requiring formal review. As the research project was clearly described in the cover letter, filling out the questionnaire by the respondents was regarded as their consent for participation in the study.

The data were analyzed using SPSS for Windows, version 16.0. Descriptive statistics were used to describe respondents’ demographic characteristics, while *T* test and one-way variance analysis or Mann–Whitney U were used for the comparison of variables. The normality of distribution of the vignettes’ RI was tested and confirmed with one-sample Kolmogorov-Smirnoftest. Moreover, Levene’s test was used for testing homogeneity of variances. In case that normality of the distribution or equality of variances was rejected, non-parametric tests were used for comparison of variables.

## Results

Of the 732 responding dentists in the German-speaking areas of Switzerland, 641 respondents had filled out the questionnaire completely, i.e. both parts - ethical judgment and prevalence estimation. The mean (SD) age of respondents was 50.65 (9.39) years, ranging between 24 and 80. There were 167 (22.8 %) female dentists in the sample, 4 (0.5 %) respondents did not specify their gender. The majority of respondents were general dentists, and the percentage of dental specialists was almost 20 %. The percentage of respondents who had graduated in Switzerland was 85, in Germany 8.7. Table 1 shows the demographics of the respondents and their academic degrees.

**Table 1:** Distributions of responding Swiss (*n* = 732) dentists (%) by gender, age and professional Factors

Characteristic	Respondents, Number (percentage)
Gender	
Female	167 (22.8)
Male	561 (76.6)
Missed	4 (0.5)
Age group (year)	
≤ 29	7 (0.9)
30-39	69 (9.4)
40-49	184 (25.1)
50-59	240 (32.8)
≥ 59	121 (16.5)
Missed	111 (15.2)
degree	
General dentist	586 (80.1)
Dental specialist	143 (19.5)
Missed	3 (0.4)

The vignette on overtreatment (or unnecessary treatment) with its corresponding five options, and the results of the two value judgments for each option – as selected by respondent categories read as follows:

*It is a short while since Dr A. has opened his/her dental private office and the patient load of the office is still low. Today, a 36-year-old patient with excellent oral health status has come to replace the fractured restoration of the lower right second molar. On examination, occlusal discolorations seem to be present in the second premolar and the first molar on the same quadrant, while clinical and bite-wing x-ray examinations reveal that there are arrested superficial decays.*

**Fig. 1:**
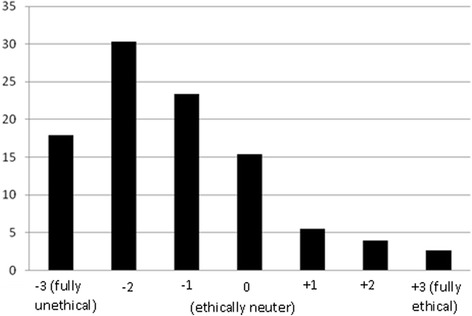
Distribution of respondents (*n* = 719) according to their ethical judgment about the first option
- *Emphasizing the necessity of restoring the second premolar and the first molar in that visit, the dentist restores them and changes the restoration of the second molar (Fig.**2**).*

- *The dentist informs the patient about the probable recurrent decays and encourages him/her to restore the second premolar and the first molar (Fig.**1**).*

**Fig. 2:**
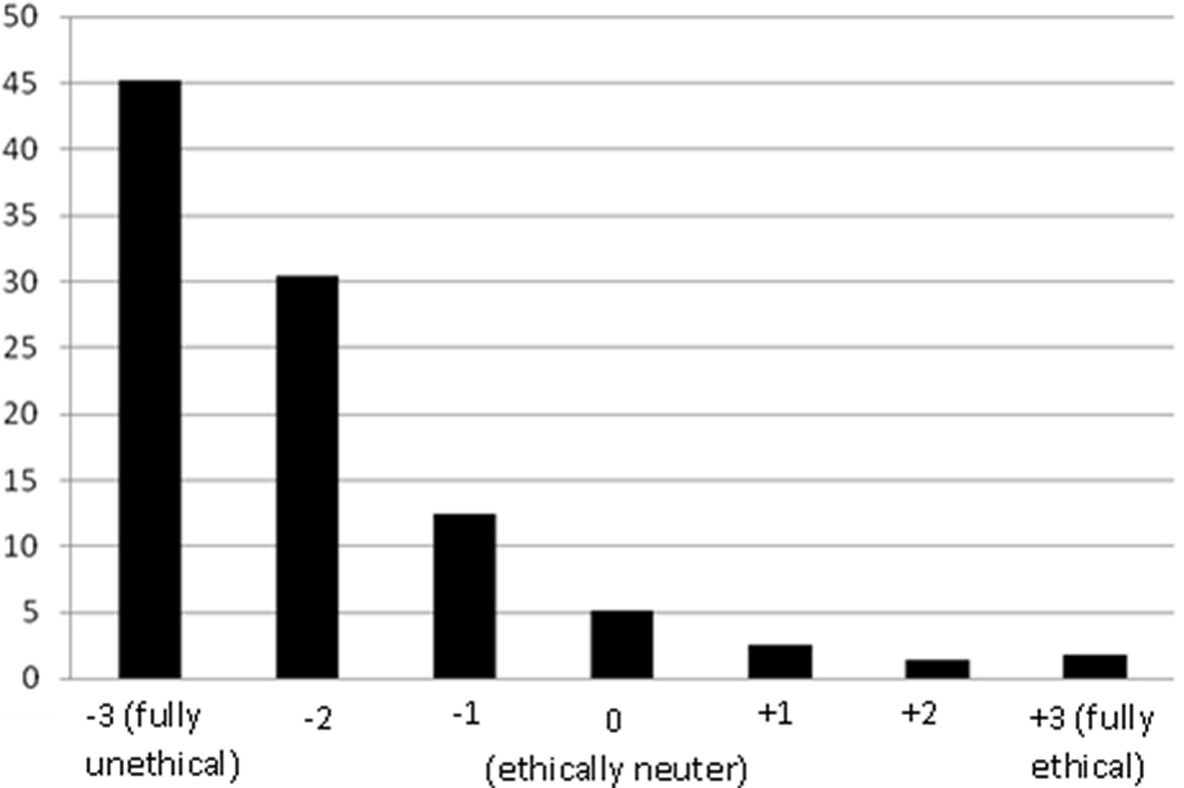
Distribution of respondents (*n* = 718) according to their ethical judgment about the second option
- *The dentist changes the restoration of the second molar and emphasizing that it is not necessary to restore the second premolar and the first molar, encourages the patient to pay attention to his/her oral health and attend check-up visits (Fig.**3**).*

**Fig. 3:**
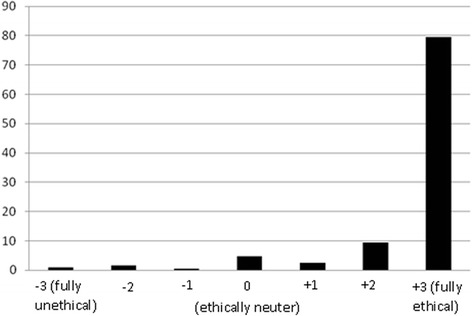
Distribution of respondents (*n* = 722) according to their ethical judgment about the third option
- *The dentist changes the restoration of the second molar and dismisses the patient without any treatment or advice on the second premolar and the first molar (Fig.**4**).*

**Fig. 4:**
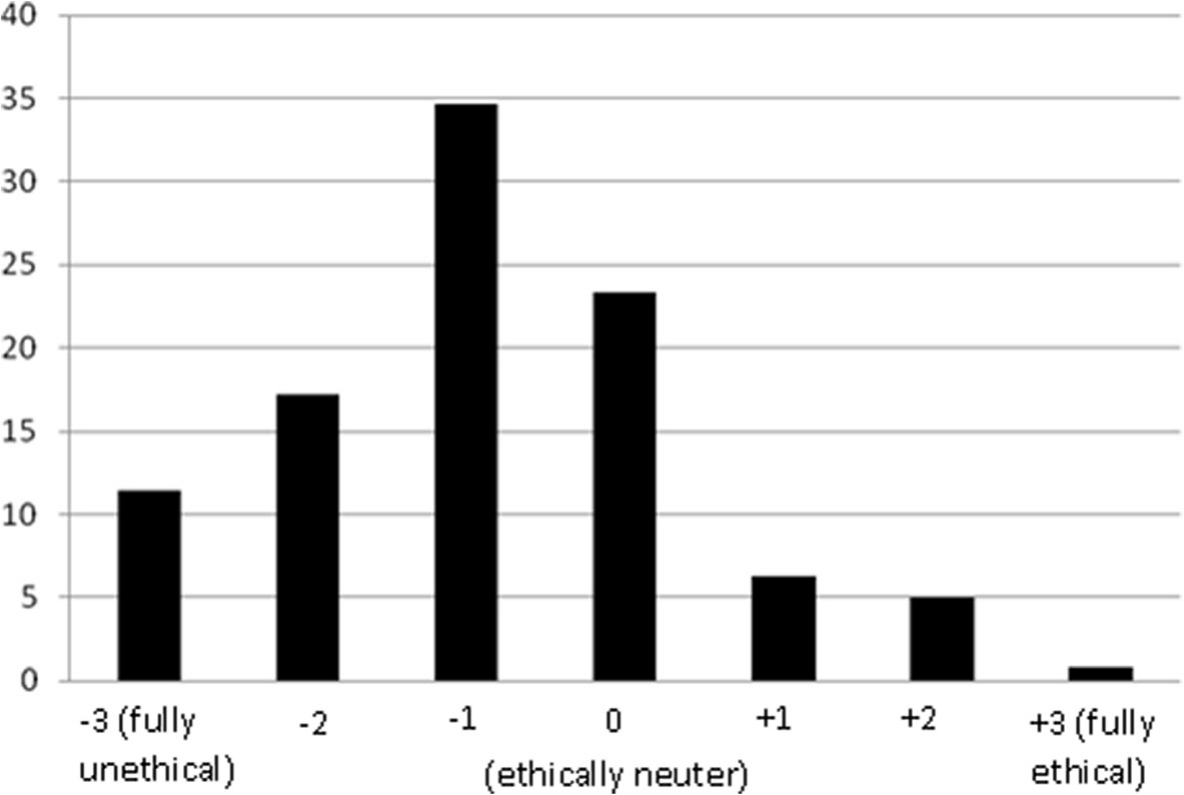
Distribution of respondents (*n* = 717) according to their ethical judgment about the fourth option
- *The dentist suggests an additional diagnostic test with CBCT*^2^*to the patient (Fig.**5**).*^3^

**Fig. 5:**
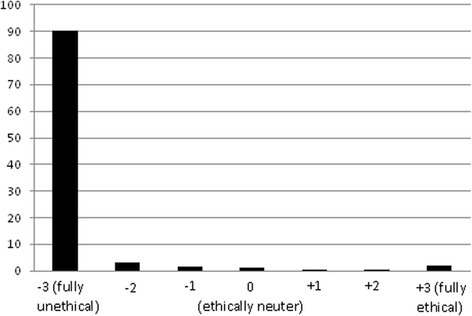
distribution of respondents (*n* = 721) according to their ethical judgment about fifth option

Table 2 shows the average percentage of dentists in Switzerland who our test sample perceived as being the likely behavior of their Swiss colleagues according to the options offered for the vignette. If the sum total of prevalence of the options in a questionnaire was not equal to one hundred, basic corrections were made by multiplying each number to 100 and dividing it by the sum total of prevalence of the options.

**Table 2:** Average percentage of dentists in Switzerland who are expected by their peers to behave according to different options of the vignette (number of respondents = 641)

	Mean	Std. Deviation
Option a	19.9	14.1
Option b	14.3	11.3
Option c	45.2	22.2
Option d	17.4	12.9
Option e	3.2	6.0

The third option was regarded as our cohort’s most expected behavior of dentists in Switzerland, in the sense that on average 45 out of 100 Swiss dentists were assumed to behave in a way described in option c; this option was regarded as representing a fully ethical behavior by the majority of respondents. Option e, however, was considered to be both, the most unethical and the least expected behavior amongst the five options of this vignette.

There was no statistically significant difference in the value judgment and prevalence estimation of the options between genders, age groups and also between general dentists and specialists.

## Discussion

The results of our study show that a sample of dentists in Switzerland believe the most likely action of their peers, in response to a situation which may end in planning overtreatment, equals the evaluation of their own perceived action - i.e. the preferred ethical option of behavior. However, the study also highlights some possible concerns in this regard. All options of the vignette, except the third one, were considered unethical by the majority of respondents. The following order of negative values was assigned to the four options from the most unethical to the least one: option e) > option b) > option a) > option d)

This ranking is based on the results of this study’s survey about the ethical sensitivity of dentists in Switzerland regarding the issue of overtreatment. Option e), considered as a fully unethical behavior by ninety percent of participants, may be seen as a critical case for overdiagnosis. It refers to the overuse of screening tests in asymptomatic individuals identifying conditions that might be indicative of problems, but could also lead to treatment for conditions that would never result in symptoms [23]. It is only one aspect of the definition of overtreatment - specifically, subjecting a patient to tests that provide little or no useful information about the patient’s condition or care [1]. Plain reliance on radiographs without employing a clinical visual-tactile method for caries lesion detection, in this example, can lead to considerable overtreatment [24]. Using diagnostic technologies with high specificity could contribute to prevention of overtreatment [25], but this topic raises another set of questions beyond this particular study.

Option b) follows next. Besides overtreatment, this option contains another ethical concept: not-being-truthful to the patient. A relevant point, here, regards the above-mentioned rank ordering of the five options; option a) is considered unethical by more respondents than option d). This may mean that the dental professionals judged unnecessary treatment to be more unethical than undertreatment or not–informing the patient about his/her probable future treatment needs. The result is surprising as informing the patient is an extremely high ranked duty also embedded in the legislation and jurisdiction and derived from the principle respect autonomy (informed consent). It might be understood, however, by referring to nonmaleficence assuming that the dentists prioritize avoiding burden (risk etc.).

The third option – the sole that was judged to be fully ethical by the majority (almost 80 %) of respondents – was at the same time considered to be the most expected behavior of dentists in Switzerland confronting this vignette. In that sense, dentists in Switzerland may be characterized as being optimistic about the ethical conduct of their colleagues when facing situations that might suggest performing unnecessary treatment. It is worth noting, however, that the respondents still seem to be concerned about the likelihood of over-treating patients by dentists. The first option, for example, which was considered unethical by the majority of respondents, was assumed to be the chosen behavior of almost one-fifth of dentists in Switzerland.

In developing this vignette to represent the ethical issue of overtreatment, we relied on a current professional norm that a non-cavitated lesion should be treated nonoperatively [26]. The dentist, as described in the vignette, used visual examination and a technological test to diagnose the suspicious lesion as an arrested decay. Another global strategy for the management of suspicious occlusal caries [27], preventive therapy, is not considered in the vignette or the listed survey options.

There is a strong tendency towards restoring enamel caries among dentists. The results of a study by Ghasemi et al. [28] indicate that dentists, however, are not yet offering patients an appropriate level of opportunity for arresting carious lesions confined to the enamel; significant evidence is emerging, though, that this possibility for arresting caries, even for high-caries cases, is highly likely [29, 30].

A survey in Washington State, based on this information, reports that adults were more likely to receive overtreatment in a dental setting, if the adult had more fillings at baseline, or if an adult’s dentist was younger, had a busy practice, advertised, charged higher fees, had less continuing education, or had a solo practice [31]. Zadik and Levin state in a paper on clinical decision making that “overtreatment among young practitioners reflects failure of undergraduate education in management of the carious lesions according to the patient’s clinical presentation and caries risk assessment rather than routinely undertaking surgical caries treatment ”[32]

Viewing the issue of overtreatment from a principle-based ethical perspective, the American Dental Association’s Principles of Ethics and Code of Professional Conduct advises: “a dentist who recommends and performs unnecessary dental services or procedures is engaged in unethical conduct” [33].

As stated by D. W. Chambers, Oral Health Care Ethics is in its infancy [34]. Exploratory studies on ethical issues of dental practice could be hardly found in the literature. To our knowledge, the most comparable case vignette in the literature on overtreatment in dental practice is case dilemma number 19 in the series presented by Hasegawa in the Texas Dental Journal [35]. The case, namely “overtreatment or unnecessary treatment”, is about an emergency patient who is scheduled by another dentist to start crowns on all of his molars next week, while there is small deficiency and no evidence of clinical and radiographic caries in his molars. Hasegawa’s focus in the discussion about the vignette was on uncertainty regarding the art and science of dentistry. This could be considered one of the central features of our case as well. Our study, however, does not focus on clinical questions such as how sure we can be in diagnosis of arrested caries and what would be the best way to manage it. Both analyzing this uncertainty and value judgment of the issue were delegated to respondents, while the researchers maintained their impartiality as far as possible.

Planning and carrying out excessive treatment was one of the five factors Christensen offered on why the public’s attitude toward the credibility of dentists may be changing [36]. Overtreatment is also one aspect of the first broad category of ethical concern within the dental profession identified by the Queensland survey, that is, problems arising from the quality of care provided by other members of the profession, including under- and over-servicing and apparently substandard treatment [37].

One common problem in a survey on ethics is the tendency of respondents to answer questions in a manner that will be viewed favorably by others, the so-called social desirability bias [38]. Furthermore, as shown in different studies, dentists’ answers to questions in a questionnaire survey do not always reflect their real practices [39, 40]. For these reasons, the questions about the own behavior of the respondent have not been included in the present questionnaire, in order to eliminate the unreal self-reporting answers from the current study. They were substituted with questions about the dentists’ estimation of what percentage of their colleagues behaves in a specific way, while confronting the situation that was described in each vignette.

According to the voluntary basis of participation in this study, it is possible that more responses were received from those who felt more confident in ethical reflection, and it might include a specific, but unknown tendency in their ethical judgment. However, it is not clear into which directions the responses tended; e.g. whether the respondents were more optimistic or rather more pessimistic about the prevailing preferences of Swiss dentists regarding ethical behavior, in comparison with those who did not fill in the questionnaire. Moreover, the nonrandom and cross-sectional nature of the current data suggests that the interpretation of results should be limited to the group examined at the time of this research. Further studies are needed to confirm validity of the questionnaire and generalisability of the results.

Regarding the length and type of the present vignettes, completing the questionnaire needed relatively long time (nearly 40 min on average). This, in addition to the high level of concentration required from the respondents, points at a high interest and motivation on their side; but it could also contribute to a low response rate. We used the following strategies to increase the response rate of the study: using stamped return envelope; designing the questionnaire to be of more interest to participants and originating the questionnaire from university, rather than other sources, such as commercial organizations, which had been shown to increase response rates to postal questionnaires [41]. These strategies were useful in reaching a response rate of nearly 30 %. Nevertheless, the significant inverse relationship between sample size and response rates [42] may explain the relatively moderate response rate of this survey despite the high number of completed questionnaires. To sum up, suggesting unnecessary treatments and procedures to patients seems to be an ethically unacceptable conduct in the eyes of a sample of dentists in Switzerland. They believe when facing a situation that is susceptible to overtreatment, the most expected behavior of their fellows is to behave in an ethical way. However, according to the currently available data, overtreatment could still be a matter of concern in the profession of dentistry.

The findings could suggest that more efforts should be made to establish ethics teaching and support in Dentistry – similar to other clinical fields with the aim to raise awareness on futile practice and questionable treatment practices and to strengthen the ethical quality of a technically highly developed field of patient care. Dentistry appears as a fruitful field for setting criteria helping to distinguish between more or less ethical options preventing the error of offering too little or too much service to patients in times of increasing need to acknowledge fair allocation of resources.

## Conclusions

The study reveals that dentists are sensitive, and dentistry is susceptible to overtreatment. Despite the moderate optimism of the respondents about the ethical conduct of their colleagues in this regard, overtreatment could be regarded as a cause for concern in dental profession.

## Footnotes

1 Ethikkommission Nordwest- und Zentralschweiz (Ethical committee of North-West and Central Switzerland)

2 Cone beam computed tomography- a medical imaging technique, which has become increasingly important in treatment planning and diagnosis in implant dentistry and interventional radiology, but not that functional for caries diagnosis

3 The vignette describes a clinical problem that according to the dental textbooks and guidelines could be followed up without any prompt intervention. Since some sorts of dental carious lesions, as what described in the scenario, are likely to be reversible, patients may be able to control them by excellent dental hygiene and without any restorative treatment. A group of dentists, however, might prefer to perform procedures in the first steps, while such decisions could be regarded as a case for unnecessary treatment
